# Functional properties of an alternative, tissue-specific promoter for rice NADPH-dependent dihydroflavonol reductase

**DOI:** 10.1371/journal.pone.0183722

**Published:** 2017-08-25

**Authors:** Joonki Kim, Hye-Jung Lee, Yu-Jin Jung, Kwon-Kyoo Kang, Wricha Tyagi, Michael Kovach, Megan Sweeney, Susan McCouch, Yong-Gu Cho

**Affiliations:** 1 Department of Crop Science, Chungbuk National University, Cheongju, Korea; 2 Department of Horticultural Life Science, Hankyong National University, Ansung, Korea; 3 Department of Plant Breeding and Genetics, Cornell University, Ithaca, New York, United States of America; National Taiwan University, TAIWAN

## Abstract

NADPH-dependent dihydroflavonol reductase (DFR) plays an important role in both anthocyanin biosynthesis and proanthocyanidin synthesis in plants. A specific and quantitative RT-PCR assay for transcription from the DFR promoter detected high expression with limited variability in rice tissues. A 440 bp minimal promoter region was identified by transfection of β-glucuronidase (GUS) reporter constructs into Jeokjinju variety. Alignment of the region with orthologous promoters revealed three conserved segments containing both bHLH (-386 to -381) and Myb (-368 to -362) binding sites. Transfection of β-glucuronidase constructs with targeted point mutations in the minimal promoter defined two sites important for promoter function to the transcription factor binding consensus sequences. The expression study showed that the bHLH binding domain (-386 to -381) is essential for DFR expression, and that a Myb binding domain (-368 to -362) is also required for full expression of the DFR gene, while the two bHLH binding domains (-104 to -99 and -27 to -22) nearest to the transcriptional start site are not necessary for DFR expression.

## Introduction

The accumulation of anthocyanins or proanthocyanidins in plant organs generally requires the presence of at least two families of regulatory proteins: Myc-type proteins containing a basic helix-loop-helix (bHLH) domain, and R2R3 Myb proteins. Genes belonging to these two families are present in numerous copies in plant genomes and encode similar proteins with varying patterns of expression that are associated with the regulation of diverse genes and pathways in different tissues and at different stages of growth [1, 2]. Studies in maize report that a member of each of these families is necessary to activate anthocyanin biosynthesis in all tissues, and a direct physical interaction between Myc (bHLH) and Myb proteins is required for activation [3]. Subsequent research has revealed significant variations on this general theme in different plant species.

In Arabidopsis and tomato, expression of the maize *Lc* (bHLH) gene induces anthocyanin production in both vegetative and reproductive organs, while in tobacco (*Nicotiana tabacum*), the same construct induces pigmentation only the floral organs [4, 5]. *Lc* orthologs from *Antirrhinum* (Delila) and *Perilla* (Myc-RP) are unable to induce anthocyanin synthesis in Arabidopsis despite high sequence similarity between these proteins [6]. Similarly, the petunia bHLH homolog, *Jaf13*, was unable to activate anthocyanin synthesis when introduced into maize bHLH mutants [7]. These results suggest that interactions between bHLH proteins and their partner Myb proteins are very specific, and the expression of these genes is restricted to certain tissues and developmental stages, which may vary by species [8]. It also highlights the importance of the combinatorial interactions between these two families of transcription factors needed to impart transcriptional activation of the flavonoid pathway.

The combinatorial interactions of bHLH and Myb proteins for regulation of the flavonoid pathway may only be part of a larger picture. Work in maize, snapdragon, petunia, and Arabidopsis has revealed that bHLH and Myb proteins are only two of several factors potentially involved in the control of flavonoid biosynthetic genes. Additional molecular factors implicated in bHLH family-mediated flavonoid biosynthetic gene regulation include WD40 proteins, such as the *TTG1* gene from Arabidopsis, the *AN11* gene from petunia, and the *PAC1* gene from maize [8–11]. While TTG1 has been shown to physically interact with a bHLH protein in Arabidopsis [10], AN11 from petunia was found to be localized in the cytosol [8]. In petunia and Arabidopsis, other regulatory factors, such as TT1, a zinc finger protein [12], and AN2, a homeodomain protein [13], have also been described where loss of function results in a complete lack of pigmentation. This further demonstrates that both a more general and a more specific understanding of bHLH-mediated transcriptional activation in diverse plant species is needed if we are to constructively harness the flavonoid and other metabolic pathways for applications in agriculture and medicine.

In this study, we focus on the regulation of proanthocyanidin accumulation in the pericarp of rice. Proanthocyanidins, or condensed tannins, give rice grains a characteristic red color, which is a feature of all wild *Oryza* species [14]. White grain color, on the other hand, is associated with domestication [15, 16]. Proanthocyanidins are polymers of flavan-3,4-diols and flavan-3-ols, and are derived from the general flavonoid pathway [17]. These and related pigments are associated with a variety of biological functions in plants, including dormancy, pest and pathogen defense, and protection against UV radiation [18].

Pericarp color in rice is conditioned by genetic variation at the *Rc* and *Rd* loci [19, 20]. The *Rc* gene encodes a Myc-type transcription factor containing a basic helix-loop-helix (bHLH) domain [15, 21]. The *Rd* gene encodes dihydroflavonol 4-reductase (DFR) and is identical to the *A* locus in rice [15, 22]. When the dominant, functional forms of *Rc* and *Rd* are present, the rice pericarp accumulates proanthocyanidins, giving the grains a red color [19]. Plants that have a nonfunctional *rd*, but a functional *Rc* have brown pericarp, while plants with non-functional alleles at *Rc* (*rc* or *rc-s*), regardless of the state of *Rd*, do not accumulate proanthocyanidins and consequently have white grains [15, 21].

This suggests that *Rd* gene expression is dependent on the presence of a functional allele at *Rc*. However, Nakai *et al*. [22] showed constant expression of DFR transcripts in both *Rc* and *rc* varieties. Furukawa *et al*. [15] also reported no translational changes of the DFR protein in either red or white-pericarp varieties. These results suggested that the *Rc* gene may not be a direct, positive regulator of *DFR* transcription, but that the *Rc*-bHLH protein may regulate an unidentified gene involved in the biosynthetic pathway of proanthocyanidins, possibly at the step between leucoanthocyanidins and proanthocyanidins [15].

To investigate whether the Rc protein directly regulates *DFR* gene expression in rice, we constructed a set of deletion mutants in the *DFR* promoter region and used a promoter::GUS reporter system to monitor *DFR* expression. Using *in silico* analysis, we first identified eight cis-DNA binding elements in the DFR promoter corresponding to both bHLH and Myb protein binding domains. We then constructed five deletion mutants targeting these core cis-elements. Our results demonstrate that the expression of DFR in rice is regulated by intact binding domains in the DFR promoter for both bHLH and Myb transcription factors, and that *Rc* alone is not sufficient for *DFR* expression.

## Results

### *DFR* expression in various tissues

**Fig 1:**
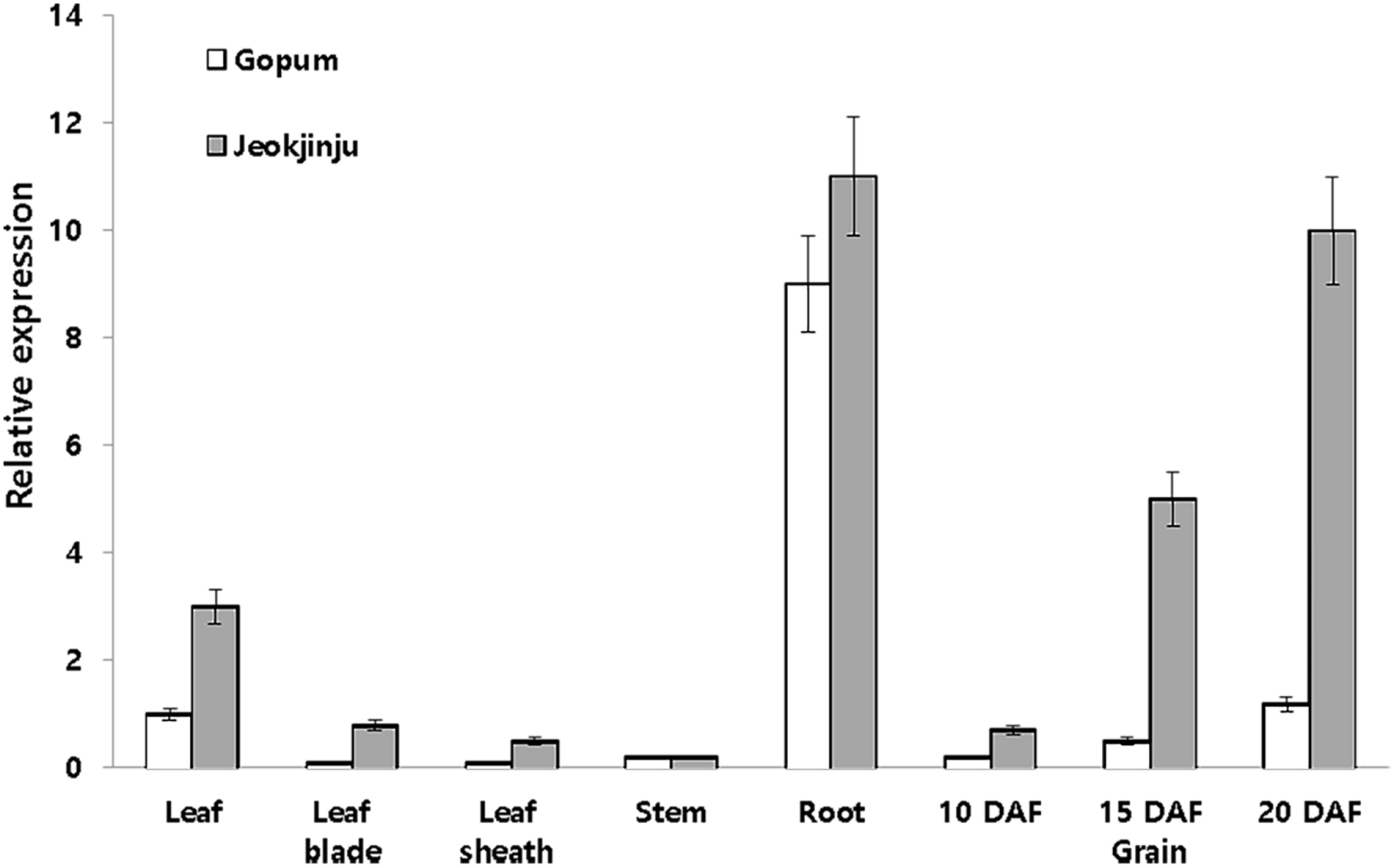
Relative mRNA expression of dihydroflavonol 4-reductase (DFR) (LOC_ Os01g44260) ortholog of Gopum (white rice) and Jeokjinju (red rice) at 20 days after flowering (DAF). The DFR expression of rice grains was examined at 10, 15, and 20 DAF during ripening stage. Beta tubulin was used as a reference gene.

*DFR* expression at 20 days after flowering (DAF) in Gopum (white pericarp) and Jeokjinju (red pericarp) varieties was observed in leaf, leaf blade, leaf sheath, stem and roots, and also in grains at 10, 15, and 20 DAF as shown in Fig 1. The DFR expression in leaf blade, leaf sheath, and stem was very low in both varieties, but the expression in leaf was 3 times higher in Jeokjinju than that in Gopum. The expression level in roots was approximately 10-fold higher than in leaves. In rice grains, DFR expression was highest at 20 DAF (ripening stage), with expression levels in Jeokjinju being about 8-fold higher than in Gopum (Fig 1).

### Analysis of cis-regulatory elements in the DFR promoter

**Fig 2:**
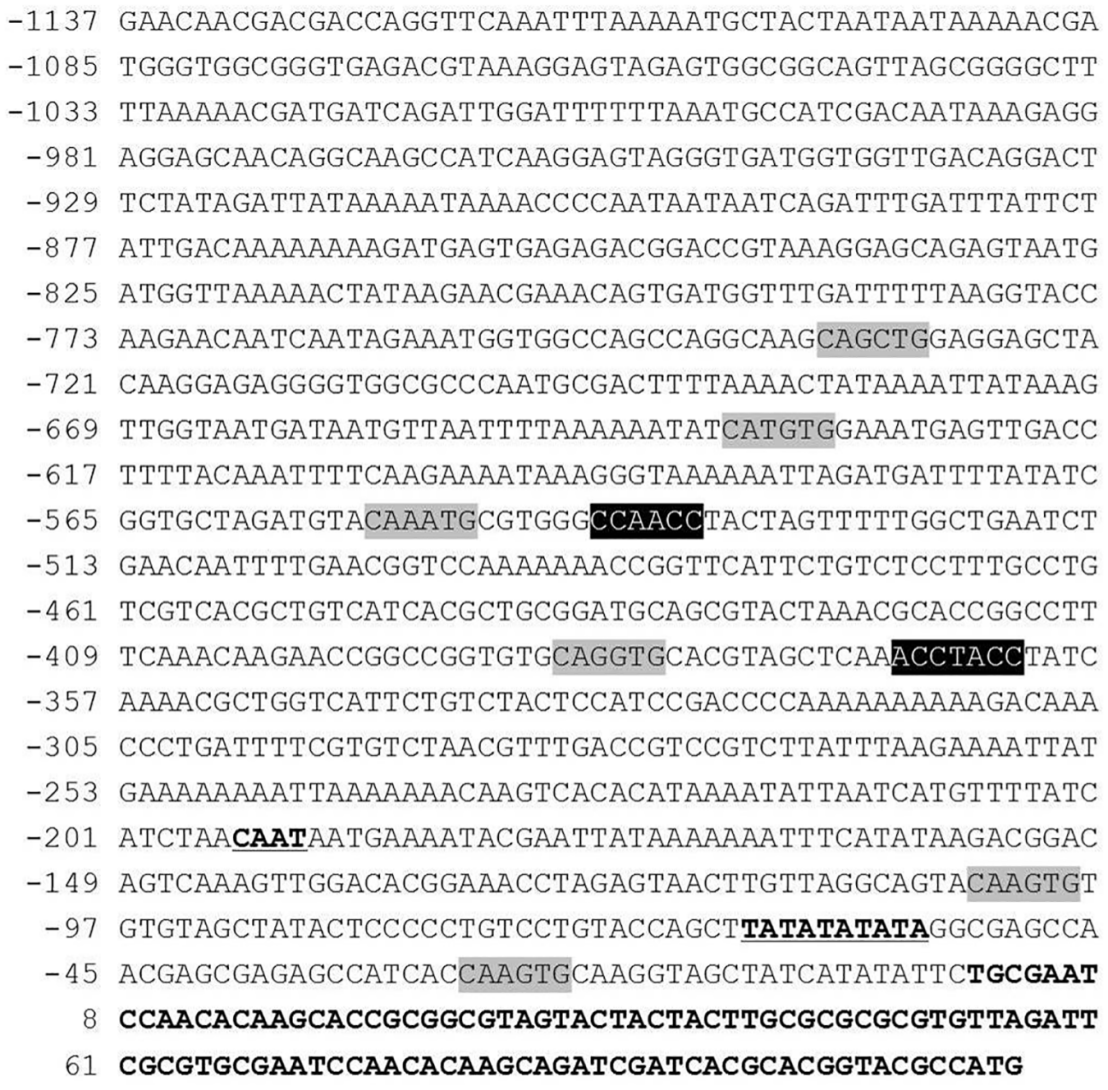
Analysis of DFR promoter region. Putative cis-acting regulatory elements detected in the promoter fragment using PlantCARE and PLACE databases are indicated. The transcription initiation site is indicated with +1. Light gray box represents the bHLH binding site and the black box represents the Myb binding site. Putative CAAT-box and TATA-box in the promoter region are indicated by the underline. The bHLH binding sites consist of CANNTG (CAGCTG, CATGTG, CAAATG, CAGGTG and CAAGTG) and Myb binding sites consist of CC(T/A)ACC (CCAACC) and AC(C/A)C(T/A)A(C/A)C (ACCTACC).

Fig 2 shows the nucleotide sequence of a 1.2 kb promoter region located immediately upstream of the *DFR* gene. The putative CAAT box and TATA box were located at -47 nt and -22 nt from the transcriptional initiation site, respectively. Six putative bHLH binding motifs and two putative Myb binding motifs were identified using *in silico* analysis (Fig 2). The consensus binding motif sequences for bHLH were CANNTG (CAGCTG, CATGTG, CAAATG, CAGGTG and CAAGTG) and Myb proteins were CC(T/A)ACC (CCAACC) and AC(C/A)C(T/A)A(C/A)C (ACCTACC). Based on the position of each selected motif, primers were designed for the construction of serial deletion constructs (Table 1).

**Table 1:** Primer sequences used in this study.

Name	Orientation	Sequences (5’- 3’)
DFR DEL1	Forward	aagcttCAACGACGACCAGGTTCAAAT
DFR DEL2	Forward	aagcttCTGGAGGAGCTACAAGGAGAGG
DFR DEL3	Forward	aagcttCTGTCTCCTTTGCCTGTCGT
DFR DEL4	Forward	aagcttCGCTGGTCATTCTGTCTACTCC
DFR DEL5	Forward	aagcttCCCTGTCCTGTACCAGCTTA
DFR DEL6	Reverse	ccatggTTCGCACGCGAATCTAACAC
1bHLHF	Forward	GGCCGGTGTGCAGGTGCACGTAGCT
1bHLHR	Reverse	AGCTACGTGCACCTGCACACCGGCC
1MybF	Forward	CACGTAGCTCAAACCTACCTATCAA
1MybR	Reverse	TTGATAGGTAGGTTTGAGCTACGTG
2bHLHF	Forward	TGTTAGGCAGTACAAGTGTGTGTAG
2bHLHR	Reverse	CTACACACACTTGTACTGCCTAACA
3bHLHF	Forward	AGAGCCATCACCAAGTGCAAGGTAGC
3bHLHR	Reverse	GCTACCTTGCACTTGGTGATGGCTCT
hpt F	Forward	atttgtgtacgcccgacagt
hpt R	Reverse	GATGTAGGAGGGCGTGGATA

### In planta expression assay of DFR::GUS

**Fig 3:**
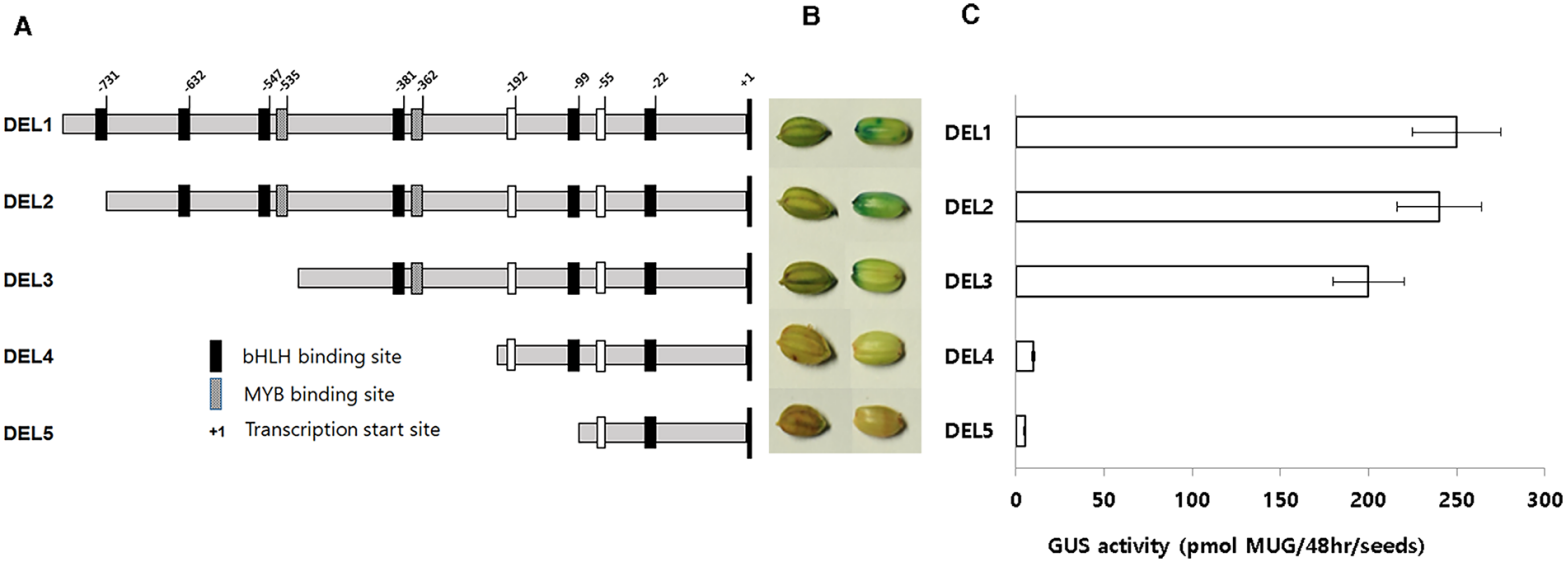
Vector construction and GUS expression of different DFR promoter deletions. Schematic representation of the size and position of each promoter construct used for transformation of rice (A), GUS expression patterns of DFR promoter constructs (B), GUS activity in deletion series of DFR promoter (C). GUS staining was detected as high levels in DEL1, DEL2, and DEL3, but not in DEL4 and DEL5.

To identify which of the core cis-elements controlled the expression of the *DFR* gene, the 1.2 kb fragment upstream of the start codon was isolated and subcloned into the GUS expression vector pCAMBIA1301 (Fig 3A). The DEL1 construct (1,203 bp) contained all six bHLH and two Myb binding motifs. DEL2 contained all DNA binding motifs except the first (most distal) bHLH (-736 to -731) motif. DEL3 had deleted the three distal bHLHs (-736 to -731, -637 to -632 and -552 to -547) and the Myb (-540 to -535), but retained the three bHLH (-386 to -381, -104 to -99 and -27 to -22) and the single MYB (-368 to -362) that were proximal to the start site of the DFR gene. DEL4 contained only the most proximal two bHLHs (-104 to -99 and -27 to -22) and DEL5 contained only bHLH (-27 to -22). These constructs were transformed into *A*. *tumefaciens* LBA4404 and then introduced into the red pericarp variety Jeokjinju (*RcRd* genotype) using *Agrobacterium*-mediated transformation. To determine whether the expression of DFR is dependent on the presence of a functional *Rc* allele, we transformed the DEL1 construct, which had a putative full-length promoter region containing all eight cis-DNA binding motifs, into a red-pericarp variety, Jeokjinju (*Rc*), and into a white-pericarp variety, Gopum (*rc*). Gus staining results showed similar expression patterns of the GUS gene in both rice varieties, regardless of which *Rc* allele was present (Fig 3B and 3C). To determine which of the motifs were essential for *DFR* expression, we introduced each individual construct, DEL1 to DEL5, into the Jeokjinju variety. T_1_ seed staining results showed that the DFR promoter drove strong expression of GUS in DEL1, DEL2 and DEL3, but expression was very weak in DEL4 and DEL5 (Fig 3B and 3C).

**Fig 4:**
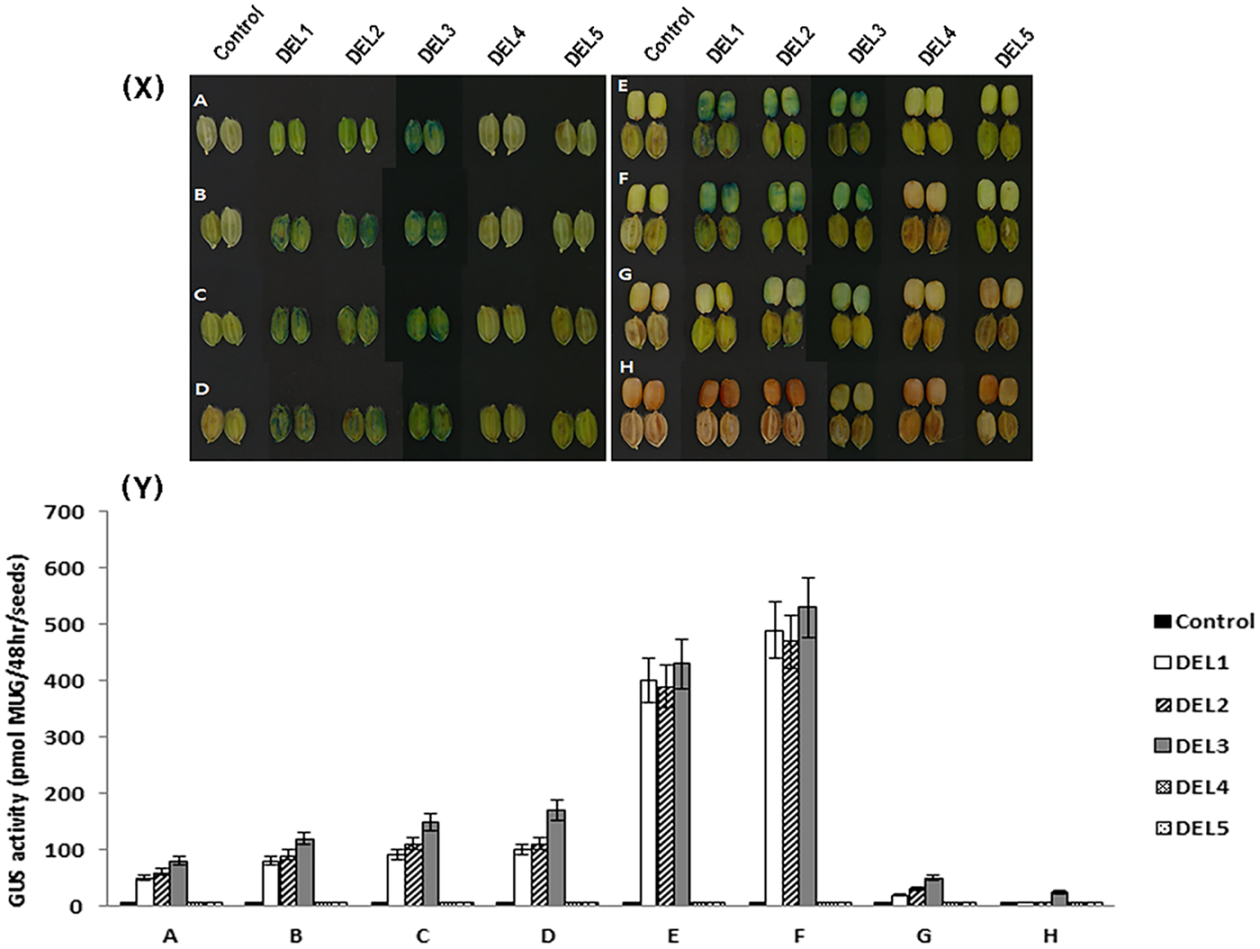
**(X) GUS staining of transgenic promoter-deletion lines**. 5 days before flowering (A), At flowering(B), 5 days after flowering (C), 10 days after flowering (D), 15 days after flowering (E), 20 days after flowering (F), 25 days after flowering (G), 30 days after flowering (H). A to D, All seeds are dehulled. E to H, Seeds without hulls are in the upper row and seeds with hulls are in the lower row. All transgenic lines are in the Jeokjinju variety. **(Y) GUS activity in all deletion series of DFR promoter was measured**. The GUS activity was highest at 20 days after flowering (F).

We also carried out GUS staining of developing grains for each of the deletion lines, from the flowering stage to the mature seed stage. GUS activity was highest at 20 DAF. DEL1, DEL2, and DEL3 transgenic lines showed constant, high levels of GUS expression in pericarp tissue from early embryogenesis to mature seed, whereas, lines carrying DEL4 and DEL5 showed very weak expression of the GUS gene (Fig 4). These results suggest that DEL3, which contains three bHLH and a single MYB binding motif located over 300 bp away from the start site of the DFR gene has the minimal configuration of *cis*-elements necessary for DFR specific expression.

### Site-directed mutagenesis of DEL3 construct

**Fig 5:**
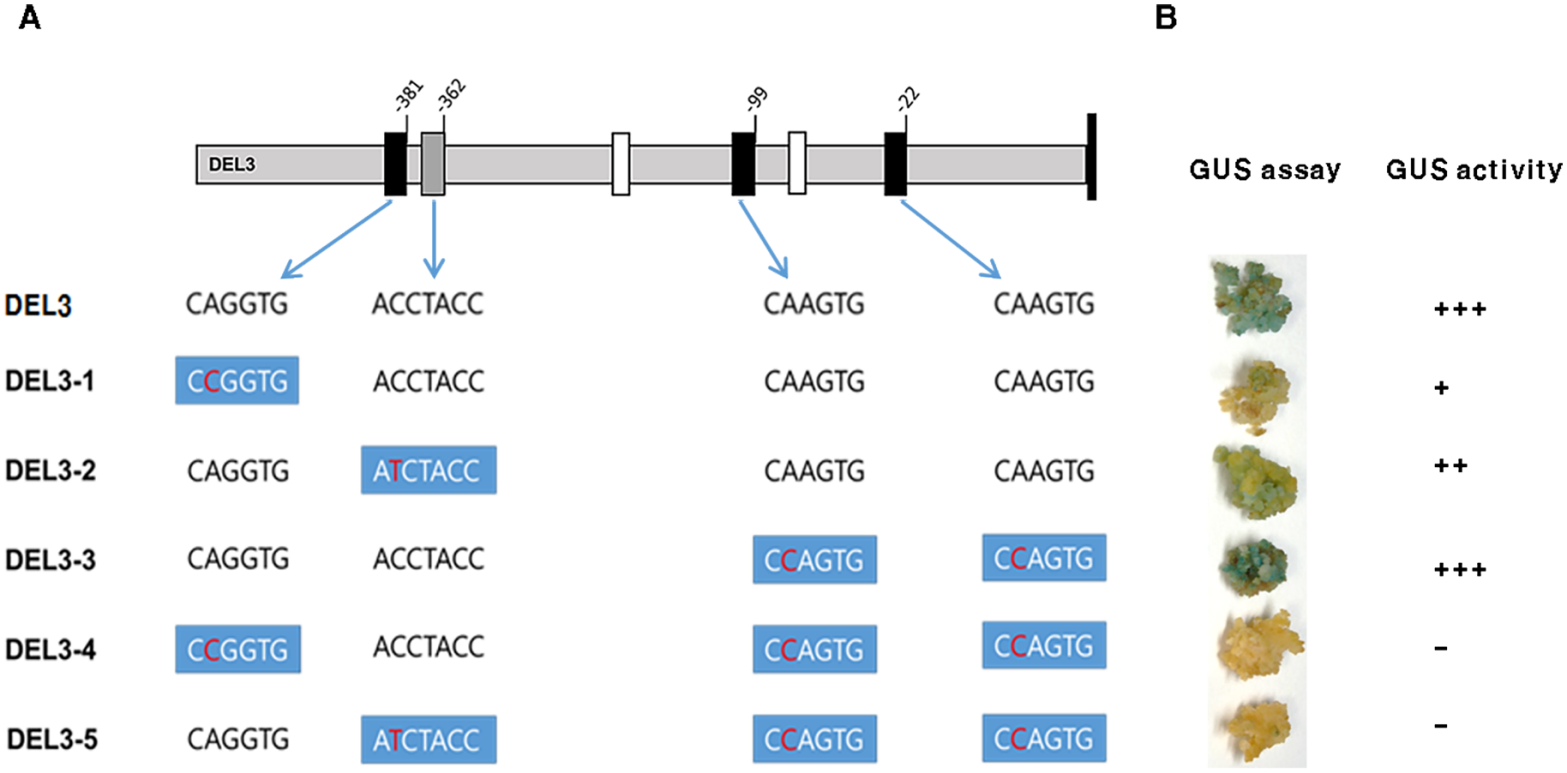
Site-directed mutagenesis of DEL3. **A.** Schematic representation of the position of each bHLH and Myb binding site and point mutation of each construct. Black boxes = bHLH binding motif; grey box = Myb binding motif. White boxes = putative CAAT and TATA box. **B.** Transient GUS expression of each mutated promoter in Jeokjinju calli.

To test more specifically the effect of the bHLH and Myb binding site mutations on DFR::GUS expression, we used site-directed mutagenesis to introduce single substitutions into three bHLH and one Myb binding site in the DEL3 promoter (Fig 5A) and then used the constructs for transient expression using the calli of the red pericarp variety, Jeokjinju (Fig 5B). DEL3-1 contains a single mutation, CAGGTG to CCGGTG, at the first bHLH (-386 to -381) binding site. DEL3-2 includes the mutation, ACCTACC to ATCTACC, at the Myb (-368 to -362) binding site, and DEL3-3 carries the mutation, CAAGTG to CCAGTG, in the last two bHLHs (-104 to -99 and -27 to -22). DEL3-4 has a single functional Myb (-368 to -362) and DEL3-5 contains a single functional bHLH (-386 to -381) binding motif (Fig 5A). Each construct containing a mutated site was transformed into *A*. *tumefaciens* LBA4404 and subsequently used to infect Jeokjinju calli to test for transient expression of each construct. In DEL3-1, infected calli expressed faintly or did not express the GUS gene at all (Fig 5B**)**. DEL3-2 showed GUS expression, but not as strong as DEL3-3, which contained mutations in the last two bHLHs. These results demonstrated that the presence of both the bHLH (-386 to -381) and the Myb (-368 to -362) binding sites are necessary for complete expression of the DFR gene (Fig 5B). DEL3-3 also confirmed that the two proximal bHLH motifs (-104 to -99 and -27 to -22) are not necessary for DFR expression, while mutations in either of the distal bHLH motifs (-386 to -381) or (-368 to -362) completely prevent DFR expression. The faint GUS expression observed in DEL3-2 suggests that a single bHLH binding site is sufficient for low levels of DFR expression, but the higher level of GUS expression in DEL3-3 demonstrates that the combination of closely linked bHLH and Myb binding sites in the DFR promoter drives higher levels of gene expression (Fig 5B).

## Discussion

Flavonoids are particularly interesting features of secondary metabolism in plants due to their roles in the production of pigments. Since plant organ pigmentation is an easily observable phenotype, the flavonoid pathway has been an ideal system for the study of metabolic pathways and gene regulation. Extensive genetic and molecular characterization of the biosynthetic and regulatory genes involved in the flavonoid pathway has provided some of the most detailed knowledge of gene interactions in plants [23].

The ability to regulate gene expression often depends on combinatorial interactions between transcription factors. In maize, a direct physical interaction between bHLH and Myb proteins is required for activation of anthocyanin biosynthetic genes [3]. Studies in mammals have shown that bHLH proteins are capable of binding to DNA cis-elements called "E-boxes" (CANNTG) in the promoters of their target genes [24–26], and these binding sites are conserved for plant bHLH proteins (sometimes referred to as "G-boxes") [27, 28]. Variability in the capacity of bHLH proteins to bind DNA directly may be a function of their interactions with other molecular factors, including Myb proteins. Myb proteins contain three α-helices, forming a helix-turn-helix structure, with the third helix making sequence-specific contacts with DNA [29, 30]. The P and C1 genes from maize encode Myb proteins that bind directly to CC(T/A)ACC sites in the A1 promoter for activation of the Al biosynthetic gene (which encodes the maize DFR) [31]. The C1 Myb protein can also bind to a variety of sequences that resemble a consensus site A(C/A)C(T/A)A(C/A)C [30]. Other consensus sites for Myb protein binding have been experimentally described in the promoters of multiple flavonoid biosynthetic genes [23, 32]. It is therefore likely that DNA binding by the bHLH, Myb, or both transcription factors together may dictate the tissue-specific activation of their target biosynthetic genes.

In this study, we dissected the role of cis-elements in the DFR promoter using a promoter::GUS fusion system to understand which elements were critical to the regulation of DFR expression. The analysis assumes that the functionally important region of the target DFR promoter is located within a 2 kb region upstream of the transcriptional start site of the gene. While it is possible that additional regulatory elements may lie further upstream, work on flavonoid pathway regulation in maize and Arabidopsis has revealed that a small region of the promoter, near the transcription start site, contains Myb and bHLH binding sites and that this region is sufficient for tissue-specific activation of several flavonoid biosynthetic genes [23, 32–35].

Using constructs containing serial deletions within the rice DFR promoter fused to the coding sequence of a GUS reporter gene, our transformation experiments showed that a promoter containing three bHLH and a single MYB binding motif located >300 bp from the transcriptional start site was capable of driving DFR expression in intact seeds, pericarp tissue and embryonic callus of the red pericarp rice variety, Jeokjinju (Rc).

Using site-directed mutagenesis, we further showed that the bHLH (-386 to -381) binding domain is essential for DFR expression, and that the Myb (-368 to -362) binding domain is also required for full expression of the DFR gene), while the two bHLHs (-104 to -99 and -27 to -22) nearest to the transcriptional start site of the DFR gene are not necessary for GUS expression in embryonic callus of the red pericarp rice variety. These findings are consistent with a previous study by Furukawa et al. [15] who suggested that *Rc* alone did not regulate the function of the DFR gene. They hypothesized that the *Rc*-bHLH gene interacts with an unidentified gene involved in the biosynthetic pathway of proanthocyanidins [15]. In this study, we identified eight cis-DNA binding elements and showed that the expression of DFR in intact seeds, pericarp tissue and embryonic callus in rice, monitored by a promoter::GUS reporter system, required the presence of both bHLH and Myb binding domains, with binding sites located at -381 bp and -362 bp, respectively, upstream of the transcriptional start site of the DFR gene.

## Materials and methods

### Plant materials and growth condition

The rice variety Jeokjinju (*Rc*, red pericarp) was used as the wild type for the generation of DFR::GUS lines. Regenerated plants were transplanted into soil medium (50% compost and 50% earth soil) in a greenhouse and acclimatized for 2 weeks. Young leaf samples were collected for genomic DNA analysis using hpt-specific primers. Plants confirmed as having the gene insert were harvested, and the T_1_ seeds were used in the subsequent planting cycle in the field. Transgenic individuals, together with the wild type plants, were sown and grown up to the T_2_ generation.

### Analysis of cis-acting regulatory DNA element of DFR promoter

Cis-acting regulatory DNA elements in the DFR promoter (1.2 kb upstream of the transcriptional initiation site) were analyzed using the online software NEW PLACE (http://www.dna.affrc.go.jp/PLACE/) [36].

### DFR promoter cloning and vector construction

A 1.2 kb region of the *DFR* promoter was amplified by PCR using DFR deletion primers (DEL1 to DEL6) and cloned into the pGEMT-easy cloning vector (Promega USA) (Table 1). All serial deletions were constructed using the pCAMBIA1301 (Cambia, Australia) binary vector, which contains a promoterless GUS gene and a CaMV 35S promoter driving the *hpt* gene encoding hygromycin B phosphotransferase. The 5 serial deletions were amplified by PCR using genomic DNA from the red pericarp variety Jeokjinju (Table 1). The 5 sets of forward primers (DFR DEL1, DFR DEL2, DFR DEL3, DFR DEL4, and DFR DEL5) used for PCR had 5’ overhang *Hind*III restriction enzyme sites and were respectively specific to 1238, 837, 581, 457, and 186 bp positions upstream of the start codon of DFR. The reverse primer, DFR DEL6 carrying the *Nco*I restriction enzyme site was designed from the 5’ UTR region of the *DFR* gene. Each PCR product was digested with *Hind*III and *Nco*I restriction enzymes (New England Biolabs, UK) and digested products were ligated into the *Hind*III and *Nco*I pre-digested pCAMBIA1301 vector to create promoter::GUS constructs. The recombinant clones were confirmed by PCR, restriction digestion analysis, and sequencing.

### Site-directed mutagenesis of the DFR promoter

For site-directed mutagenesis, primers used to mutagenize the regulatory elements were designed using the web program (http://labtools.stratagene.com/QC) (Table 1) and purified by polyacrylamide gel electrophoresis. The mutant strand synthesis reaction mixture (50uL) contained 1X PfuUltra^™^ buffer, 200 uM each dNTPs, about 10 ng of template plasmid, 2.5 unit of PfuUltra^™^ (Stratagene, USA), and 125 ng of each primers. The PCR runs were 20 cycles with each cycle at 95ycle at 95sec, 55°C for 30 sec, and 68°C for 20 min to allow for the completion of the polymerizations. After mutant strand synthesis, PCR products were digested with 10 units of *Dpn*I restriction enzyme at 377the completion of the polymerizations. After muL of *Dpn*I digested DNA was transformed into DH5α, and five independent clones were sequenced.

### Generation of transgenic plants and histochemical staining of GUS expression

The five promoter GUS constructs were electroporated into *A*. *tumefaciens* LBA4404 and introduced into pre-soaked rice seed using the method of Lee et al. [37] with minor modifications. Intact seeds, excised young pericarp tissue, and embryogenic calli originating from the red pericarp variety Jeokjinju and transgenic plants containing each deletion construct were used to estimate GUS expression. Histochemical GUS assays were performed as described by Jefferson et al. [38], and relative GUS expression was estimated using a fluorescence spectrophotometer (Bio-Rad, USA) with the excitation and emission wavelengths set at 360 and 465 nm, respectively. The GUS reaction solution consisted of sodium phosphate (Na_4_PO_4_, pH7.0), Triton-X-100, potassium ferricyanide (K_3_FeCN_6_), potassium ferrocyanide (K_4_FeCN_6_), and 5-bromo-4-chloro-3-indolyl-ferricyanide (Ksphagluc). The samples were vacuum-infiltrated using a GUS reaction solution and incubated at 37°C for 24 hr. The reaction was stopped by adding 70% (v/v) ethanol, and the pigments and chlorophylls were removed by repeated ethanol treatment.

### Analysis of GUS gene expression using RT-PCR

Total RNA was extracted from leaves collected from each transgenic rice line and the control Jeokjinju variety using the RNeasy Plant Mini Kit (QIAGEN, Maryland, USA). The relative purity and concentration of extracted RNA was estimated using a NanoDrop-1000 spectrophotometer (NanoDrop Technologies, Inc. Wilmington, USA), and RNA samples were then stored in a freezer at -80°C. Total RNAs were purified using *DNase* I, and first-strand cDNA synthesis was performed by reverse transcription of mRNA using Oligo (dT)_20_ primer and SuperScript^™^ III Reverse Transcriptase (Invitrogen, USA). The specific sequences of the primer pairs used in a semi-quantitative reverse transcription-PCR (RT-PCR) are GUS-F (5’ -AGTGTGGGTCAATAATCAGGAAGT-3’) and GUS-R (5AATCATGGAAGTAAGACTGCTTTTTCT-3’). For normalization of the quantitative RT-PCR reaction, the actin gene was used as an internal control and amplified using the primers ACT-F (5’-TGTATGCCAGTGGTCGTACC-3’) and ACT-R (5’-CCAGCAAGGTCGAGACGAA-3’) [39].

### Expression of DFR gene in different tissues

Rice seeds of Gopum (white rice) and Jeokjinju (red rice) were dehulled and surface sterilized before plating in half strength MS media. After ten days of growth, leaf, stem and root tissues were harvested for RNA extraction. TRIzol^®^ Reagent was used to isolate high quality total RNA. First-strand cDNA was synthesized with an oligo(dT) primer. The primers used to detect DFR expression by a semi-quantitative reverse transcription-PCR (RT-PCR) were 5’- ACTTCTCGTCGTGGAAGCTC-3’ and 5’-GAAGCCCCTTCTCCCTGC-3’.

## References

[1] Feller A, Machemer K, Braun EL, Grotewold E. Evolutionary and comparative analysis of MYB and bHLH plant transcription factors. Plant J. 2011;66(1):94–116. doi: 10.1111/j.1365-313X.2010.04459.x 21443626 10.1111/j.1365-313X.2010.04459.x

[2] Koes R, Verweij W, Quattrocchio F. Flavonoids: a colorful model for the regulation and evolution of biochemical pathways. Trends Plant Sci. 2005;10(5):236–42. doi: 10.1016/j.tplants.2005.03.002 15882656 10.1016/j.tplants.2005.03.002

[3] Goff SA, Cone KC, Chandler VL. Functional analysis of the transcriptional activator encoded by the maize B gene: evidence for a direct functional interaction between two classes of regulatory proteins. Genes Dev. 1992; 6: 864–875. 1577278 10.1101/gad.6.5.864

[4] Lloyd AM, Walbot V, Davis RW. Arabidopsis and Nicotiana anthocyanin production activated by maize regulators R and C1. Science. 1992; 258: 1773–1775. 1465611 10.1126/science.1465611

[5] Mooney M, Desnos T, Harrison K, Jones J, Carpenter R, Coen E. Altered regulation of tomato and tobacco pigmentation genes caused by the delila gene of Antirrhinum. Plant Journal. 1995; 7: 333–339.

[6] Gong ZZ, Yamagishi E, Yamazaki M, Saito K. A constitutively expressed Myc-like gene involved in anthocyanin biosynthesis from *Perilla frutescens*: molecular characterization, heterologous expression in transgenic plants and transactivation in yeast cells. Plant Mol Biol. 1999; 41: 33–44. 10561066 10.1023/a:1006237529040

[7] Quattrocchio F, Wing JF, van der Woude K, Mol JN, Koes R. Analysis of bHLH and MYB domain proteins: species-specific regulatory differences are caused by divergent evolution of target anthocyanin genes. Plant J. 1998; 13: 475–488. 9680994 10.1046/j.1365-313x.1998.00046.x

[8] de Vetten N, Quattrocchio F, Mol J, Koes R. The an11 locus controlling flower pigmentation in petunia encodes a novel WD-repeat protein conserved in yeast, plants, and animals. Genes Dev. 1997; 11: 1422–1434. 9192870 10.1101/gad.11.11.1422

[9] Bharti AK, Khurana JP. Molecular characterization of transparent testa (tt) mutants of *Arabidopsis thaliana* (ecotype Estland) impaired in flavonoid biosynthetic pathway. Plant Science. 2003; 165: 1321–1332.

[10] Carey CC, Strahle JT, Selinger DA, Chandler VL. Mutations in the pale aleurone color1 regulatory gene of the Zea mays anthocyanin pathway have distinct phenotypes relative to the functionally similar TRANSPARENT TESTA GLABRA1 gene in Arabidopsis thaliana. Plant Cell. 2004; 16: 450–464. doi: 10.1105/tpc.018796 14742877 10.1105/tpc.018796 PMC341916

[11] Sompornpailin K, Makita Y, Yamazaki M, Saito K. A WD-repeat-containing putative regulatory protein in anthocyanin biosynthesis in Perilla frutescens. Plant Mol Biol. 2002; 50: 485–495. 12369624 10.1023/a:1019850921627

[12] Sagasser M, Lu GH, Hahlbrock K, Weisshaar B. *A*. *thaliana* TRANSPARENT TESTA 1 is involved in seed coat development and defines the WIP subfamily of plant zinc finger proteins. Genes Dev. 2002; 16: 138–149. doi: 10.1101/gad.212702 11782451 10.1101/gad.212702 PMC155310

[13] Kubo H, Peeters AJ, Aarts MG, Pereira A, Koornneef M. ANTHOCYANINLESS2, a homeobox gene affecting anthocyanin distribution and root development in Arabidopsis. Plant Cell. 1999; 11: 1217–1226. 10402424 10.1105/tpc.11.7.1217 PMC144283

[14] Oki T, Masuda M, Kobayashi M, Nishiba Y, Furuta S, Suda I, et al Polymeric procyanidins as radical-scavenging components in red-hulled rice. J Agric Food Chem. 2002; 50: 7524–7529. 12475265 10.1021/jf025841z

[15] Furukawa T, Maekawa M, Oki T, Suda I, Iida S, Shimada H, et al The Rc and Rd genes are involved in proanthocyanidin synthesis in rice pericarp. Plant J. 2007; 49: 91–102. doi: 10.1111/j.1365-313X.2006.02958.x 17163879 10.1111/j.1365-313X.2006.02958.x

[16] Sweeney MT, Thomson MJ, Cho YG, Park YJ, Williamson SH, Bustamante CD, et al Global dissemination of a single mutation conferring white pericarp in rice. PLoS Genet. 2007; 3: e133 doi: 10.1371/journal.pgen.0030133 17696613 10.1371/journal.pgen.0030133 PMC1941752

[17] Xie DY, Dixon RA. Proanthocyanidin biosynthesis—still more questions than answers? Phytochemistry. 2005; 66: 2127–2144. doi: 10.1016/j.phytochem.2005.01.008 16153412 10.1016/j.phytochem.2005.01.008

[18] Winkel-Shirley B. Flavonoid biosynthesis. A colorful model for genetics, biochemistry, cell biology, and biotechnology. Plant Physiol. 2001; 126: 485–493. 11402179 10.1104/pp.126.2.485 PMC1540115

[19] Kato S, Ishikawa J. On the ingeritance of the pigment of red rice. Jap. J. Genet. 1921; 1: 1–7.

[20] Kinoshita T. Linkage mapping using mutant genes in rice. Rice Genet. Newsl. 1998; 15: 13–74.

[21] Sweeney MT, Thomson MJ, Pfeil BE, McCouch SR. Caught red-handed: Rc encodes a basic helix-loop-helix protein conditioning red pericarp in rice. Plant Cell. 2006; 18: 283–294. doi: 10.1105/tpc.105.038430 16399804 10.1105/tpc.105.038430 PMC1356539

[22] Nakai K, Inagaki Y, Nagata H, Miykzaki C Iida S. Molecular Characterization of the Gene for Dihydroflavonol 4-Reductase of Japonica Rice Varieties. Plant Biotechnology. 1998; 15: 221–225.

[23] Tuerck JA, Fromm ME. Elements of the maize A1 promoter required for transactivation by the anthocyanin B/C1 or phlobaphene P regulatory genes. Plant Cell. 1994; 6: 1655–1663. doi: 10.1105/tpc.6.11.1655 7827497 10.1105/tpc.6.11.1655 PMC160551

[24] Atchley WR, Terhalle W, Dress A. Positional dependence, cliques, and predictive motifs in the bHLH protein domain. J Mol Evol. 1999; 48: 501–516. 10198117 10.1007/pl00006494

[25] Brownlie P, Ceska T, Lamers M, Romier C, Stier G, Teo H, et al The crystal structure of an intact human Max-DNA complex: new insights into mechanisms of transcriptional control. Structure. 1997; 5: 509–520. 9115440 10.1016/s0969-2126(97)00207-4

[26] Ellenberger T, Fass D, Arnaud M, Harrison SC. Crystal structure of transcription factor E47: E-box recognition by a basic region helix-loop-helix dimer. Genes Dev. 1994; 8: 970–980. 7926781 10.1101/gad.8.8.970

[27] Kawagoe Y, Murai N. Four distinct nuclear proteins recognize in vitro the proximal promoter of the bean seed storage protein beta-phaseolin gene conferring spatial and temporal control. Plant J. 1992; 2: 927–936. 1302641 10.1046/j.1365-313x.1992.t01-6-00999.x

[28] Loulergue C, Lebrun M, Briat JF. Expression cloning in Fe2+ transport defective yeast of a novel maize MYC transcription factor. Gene. 1998; 225: 47–57. 9931428 10.1016/s0378-1119(98)00531-9

[29] Grotewold E, Sainz MB, Tagliani L, Hernandez JM, Bowen B, Chandler VL. Identification of the residues in the Myb domain of maize C1 that specify the interaction with the bHLH cofactor R. Proc Natl Acad Sci U S A. 2000; 97: 13579–13584. doi: 10.1073/pnas.250379897 11095727 10.1073/pnas.250379897 PMC17618

[30] Sainz MB, Grotewold E, Chandler VL. Evidence for direct activation of an anthocyanin promoter by the maize C1 protein and comparison of DNA binding by related Myb domain proteins. Plant Cell. 1997; 9: 611–625. doi: 10.1105/tpc.9.4.611 9144964 10.1105/tpc.9.4.611 PMC156943

[31] Grotewold E., Drummond B.J., Bowen B. and Peterson T. The myb-homologous P gene controls phlobaphene pigmentation in maize floral organs by directly activating a flavonoid biosynthetic gene subset. Cell. 1994; 76: 543–553. 8313474 10.1016/0092-8674(94)90117-1

[32] Lesnick ML, Chandler VL. Activation of the maize anthocyanin gene a2 is mediated by an element conserved in many anthocyanin promoters. Plant Physiol. 1998; 117: 437–445. 9625696 10.1104/pp.117.2.437 PMC34963

[33] Bodeau JP, Walbot V. Structure and regulation of the maize Bronze2 promoter. Plant Mol Biol. 1996; 32: 599–609. 8980512 10.1007/BF00020201

[34] Debeaujon I, Nesi N, Perez P, Devic M, Grandjean O, Caboche M, et al Proanthocyanidin-accumulating cells in Arabidopsis testa: regulation of differentiation and role in seed development. Plant Cell. 2003; 15: 2514–2531. doi: 10.1105/tpc.014043 14555692 10.1105/tpc.014043 PMC280558

[35] Roth BA, Goff SA, Klein TM, Fromm ME. C1- and R-dependent expression of the maize Bz1 gene requires sequences with homology to mammalian myb and myc binding sites. Plant Cell. 1991; 3: 317–325. doi: 10.1105/tpc.3.3.317 1840914 10.1105/tpc.3.3.317 PMC160002

[36] Higo K, Ugawa Y, Iwamoto M, Korenaga T. Plant cis-acting regulatory DNA elements (PLACE) database: 1999. Nucleic Acids Res. 1999; 27: 297–300. 9847208 10.1093/nar/27.1.297 PMC148163

[37] Lee HJ, Abdula SE, Jee MG, Jang DW, Cho YG. High-efficiency and Rapid Agrobacterium-mediated genetic transformation method using germinating rice seeds. Journal of Plant Biotechnolgy. 2011; 38: 251–257.

[38] Jefferson RA, Kavanagh TA, Bevan MW. GUS fusions: beta-glucuronidase as a sensitive and versatile gene fusion marker in higher plants. EMBO J. 1987; 6: 3901–3907. 3327686 10.1002/j.1460-2075.1987.tb02730.x PMC553867

[39] Reece K.S., McElroy D. and Wu R. Genomic nucleotide sequence of four rice (*Oryza sativa*) *actin* genes. *Plant Mol*. *Biol*., 1990; 14(4): 621–624. 2102841 10.1007/BF00027508

